# Punicalagin Alleviates Diabetic Kidney Injury: Potential Involvement of SESN2-Related Mitophagy and Changes in Pyroptosis-/Ferroptosis-Related Indicators

**DOI:** 10.3390/nu18193192

**Published:** 2026-09-27

**Authors:** Yue Huang, Jiazhen He, Yizhen Ma, Ziyi You, Yuhan Zhang, Rou Zhang, Xiuying Tan, Lina Yang

**Affiliations:** Xiangya School of Public Health, Central South University, Changsha 410013, China

**Keywords:** punicalagin, SESN2, diabetic kidney disease, mitophagy, pyroptosis, ferroptosis

## Abstract

Background/Objectives: Diabetic kidney disease (DKD) is a progressive nephropathy associated with excessive pyroptosis and ferroptosis. Impaired renal mitophagy has been implicated in inflammation and lipid peroxidation in DKD; however, it remains unclear whether and how it modulates pyroptosis and ferroptosis. Punicalagin (PU), a natural polyphenol with potent anti-inflammatory and antioxidant activities, has been shown to improve DKD, yet its underlying mechanism requires further elucidation. This study aimed to investigate whether PU alleviates pyroptosis- and ferroptosis-related indicators in diabetes-associated renal injury in association with SESN2-related mitophagy, using both in vivo and in vitro models. Methods: We established a diabetic mouse model using a high-fat diet and streptozotocin (STZ) injection. After successful modeling, we measured serum blood urea nitrogen (BUN) and creatinine (CREA), and stained renal tissues with HE, Masson, and PASM for pathological scoring. Protein expression of kidney injury molecule-1 (KIM-1), pyroptosis-related (caspase-1, IL-18), ferroptosis-related (FTH1, ACSL4), mitophagy-related (PINK1, Parkin, p62, LC3-II/I), and SESN2 were quantified by Western blotting. In high-glucose-induced HK-2 cells, glucose uptake was assessed by 2-NBDG, pyroptotic/ferroptotic morphology by electron microscopy, and TOM20/LC3 co-localization by confocal microscopy. Furthermore, Mitochondrial Division Inhibitor 1 (Mdivi-1) was used to probe the potential involvement of mitophagy in the effects of PU on pyroptosis- and ferroptosis-related markers, and transfection of *SESN2* siRNA was used to investigate whether the effects of PU on mitophagy-related signaling are associated with SESN2 expression. Results: Diabetic mice exhibited renal histological abnormalities, elevated BUN/CREA, and increased KIM-1 expression. Additionally, pyroptosis- and ferroptosis-related proteins were dysregulated, while mitophagy-related proteins and SESN2 were downregulated. PU treatment significantly reversed these abnormalities, which were confirmed in high-glucose-treated HK-2 cells. In the high-glucose-induced HK-2 cell model, treatment with Mdivi-1 attenuated the effects of PU on pyroptosis- and ferroptosis-related markers. In parallel, knockdown of *SESN2* by siRNA partially reversed the PU-induced changes in mitophagy-related protein expression. Conclusions: PU may modulate mitophagy-related signaling in association with SESN2, accompanied by changes in pyroptosis- and ferroptosis-related markers in diabetes-associated renal injury. This study provides preliminary evidence for the renoprotective effect of PU against DKD, potentially involving SESN2-related mitophagy and the concurrent regulation of pyroptosis- and ferroptosis-related markers in renal cells. Collectively, PU warrants further investigation as a candidate compound for DKD.

## 1. Introduction

Diabetic kidney disease (DKD) is one of the common complications of diabetes. Characterized by persistent proteinuria and declining glomerular filtration rate, DKD is the leading cause of chronic kidney disease and end-stage renal disease worldwide. According to the latest report, *Diabetes and Kidney Disease 2023*, issued by the International Diabetes Federation (IDF), the number of new cases of chronic kidney disease caused by type 2 diabetes globally increased from 1.4 million to 2.4 million from 1990 to 2017, a 74% increase. About 30% to 40% of individuals with diabetes progress to DKD [1]. Based on the large number of diabetes patients, there is great public health significance in preventing and delaying the onset and development of DKD.

The pathogenesis of DKD is complex and remains incompletely understood. Current studies have identified disorders in cell death modes, such as pyroptosis with inflammation as the core and ferroptosis marked by iron accumulation and lipid peroxidation, as an important part of the pathological process of DKD [2]. Pyroptosis, a type of programmed cell death with inflammatory properties, is accompanied by the secretion of numerous pro-inflammatory cytokines, which consequently results in chronic kidney inflammation. Cysteinyl-aspartate acid protease-1 (caspase-1) is a key molecule in the classical pyroptosis pathway. Caspase-1 activation leads to the release of interleukin-1β (IL-1β) and interleukin-18 (IL-18) into the extracellular space, triggering an inflammatory response [3]. Ferroptosis is defined as a cell death modality driven by iron overload and heightened lipid peroxidation. Excess intracellular Fe^2+^ produces hydroxyl radicals through Fenton chemistry, initiating lipid peroxidation and culminating in ferroptotic cell death [4]. Among them, acyl-CoA synthetase long-chain family member 4 (ACSL4) is a key participant in ferroptosis, which can oxidize free polyunsaturated fatty acids into toxic lipid peroxides [5]. Inhibition of caspase-1 activation and attenuation of renal inflammation [6,7,8], as well as reduction in lipid peroxidation levels [9,10,11], are both beneficial for preserving renal function and alleviating kidney injury. Therefore, understanding how pyroptosis- and ferroptosis-related signaling is regulated may provide insights into the mechanisms underlying DKD progression.

Previous studies have shown that accumulation of fragmented mitochondria has been observed in both the renal cortex of patients with DKD and streptozotocin (STZ)-induced diabetic mice, suggesting that mitochondrial clearance mechanisms in the kidney may be impaired under diabetic conditions [12,13]. Damaged mitochondria are selectively transported to lysosomes for degradation through the autophagy system to maintain mitochondrial quality and quantity, a process known as mitophagy. Among them, PTEN-induced kinase 1 (PINK1) and Parkin are currently the most extensively studied mitophagy pathways [14]. Recent studies indicate that mitophagy plays a significant role in regulating both pyroptosis and ferroptosis [15]. Enhancing the mitophagy signaling pathway can attenuate inflammatory responses, iron accumulation, lipid peroxidation, and other pathological processes in renal cells, thereby effectively inhibiting pyroptosis and ferroptosis [16,17]. However, it remains unclear whether mitophagy is involved in the concurrent regulation of pyroptosis and ferroptosis in renal tubular cells.

Sestrin2 (SESN2) is a stress-induced protein with high evolutionary conservation. It plays a crucial role in controlling mitochondrial function, notably by improving mitophagy [18]. It has been demonstrated that SESN2 enhances mitophagy by promoting Parkin translocation. SESN2 downregulation impairs the association between Beclin1 and Parkin, reducing mitophagy levels [19]. Therefore, SESN2 may act as a potential upstream modulator of mitophagy-related signaling in DKD.

Punicalagin (PU) accounts for about 50% of the polyphenol content in pomegranate peel and is the most important polyphenolic active substance [20]. Naturally extracted PU exhibits a range of physiological activities, including anti-inflammatory and antioxidant effects, and is readily digested and absorbed by the human body [21]. Our previous study showed that PU alleviates DKD by inhibiting TXNIP/NLRP3 pathway-mediated pyroptosis, mainly by improving renal function impairment, alleviating renal pathological changes, and reducing renal mitochondrial damage [22]. However, based on existing studies and our preliminary findings, whether PU can regulate pyroptosis- and ferroptosis-related indicators in diabetes-associated renal injury in association with SESN2-related mitophagy has not yet been reported.

Therefore, this study established an in vitro and in vivo model to investigate whether PU attenuates pyroptosis- and ferroptosis-related indicators in diabetes-associated renal injury in association with SESN2-related mitophagy, aiming to provide preliminary evidence for the renoprotective effects of PU and to explore this mechanism, and to provide a basis for further investigation of PU in DKD. 

## 2. Materials and Methods

### 2.1. Animals and Experimental Design

Twenty-four 8-week-old male C57BL/6 mice were obtained from Central South University (Changsha, China), acclimated for one week, and housed in environmentally controlled cages (temperature at 24 ± 2 °C, humidity at 50 ± 10%, 12 h light/dark cycles) with unrestricted access to food and water. Mice were randomly divided into a normal chow group (CON, *n* = 8, 10 kcal% fat) and a high-fat-diet group (HFD, *n* = 16, 60 kcal% fat). The basic diet (#D12450J) and high-fat diet (#D12492) were obtained from Research Diets, Inc. (New Brunswick, NJ, USA). To generate the high-fat diet/STZ diabetes model, HFD mice were fed a high-fat diet for 8 weeks. Then, mice received a single intraperitoneal injection of streptozotocin (STZ) at 100 mg/kg (freshly dissolved in sodium citrate buffer, pH 4.5) after a 12 h fast. CON mice received an equal volume of buffer. Diabetes was defined as fasting blood glucose (FBG) ≥ 11.1 mmol/L. Diabetic mice were then randomly assigned to the diabetes model group (DM, *n* = 8) and the PU treatment group (DM + PU, *n* = 8). PU (purity ≥ 98%, 20 mg/kg/day) was administered by gavage for 10 weeks, while the other groups received distilled water. PU was obtained from Chengdu Herbpurify Co., Ltd. (65995-63-3; Chengdu, China). Body weight was recorded weekly. During administration, water intake, body weight, and blood glucose levels of the mice were monitored. At the end of the experiment, mice were anesthetized with an intraperitoneal injection of 50 mg/kg of pentobarbital sodium solution. Blood was then collected from the orbital sinus. After that, the mice were euthanized by cervical dislocation, and the kidneys were immediately excised and weighed. A portion of each renal tissue was fixed in 4% paraformaldehyde for histological examination, and the remaining kidney tissue was stored frozen at −80 °C for Western blot detection. All animal experiments were performed in accordance with the protocol approved by the Institutional Animal Care and Use Committee of Central South University.

### 2.2. Biochemical Measurements in Serum

Blood urea nitrogen (BUN, mmol/L) and blood creatinine (CREA, μmol/L) levels were measured using commercial assay kits, both obtained from the Nanjing Jiancheng Bioengineering Institute (Nanjing, China).

### 2.3. Histological Analysis

Kidney tissue was fixed in 4% paraformaldehyde, embedded in paraffin, and sectioned. After dewaxing and washing, hematoxylin–eosin staining (HE), Masson’s Trichrome staining, and Periodic Acid-Schiff Methenamine silver staining (PASM) were performed. In order to quantify renal injury, HE-stained sections were scored using the Paller method [23] to assess the degree of renal tubular injury (Table S1). ImageJ (version 1.6.0; NIH, Bethesda, MD, USA) was used for semi-quantitative analysis of MASSON- and PASM-stained renal sections to determine the collagen volume fraction (CVF) and the PASM-positive area percentage.

### 2.4. Cell Culture and Treatment

The human proximal tubule epithelial cell line (HK-2) was obtained from the Xiangya Central Laboratory Cell Bank at Central South University (Changsha, China). Cells were cultured in DMEM/F12 supplemented with 10% fetal bovine serum and incubated in a humidified incubator at 37 °C with 5% CO_2_. The cells were divided into a control group (CON, Glu: 17.5 mM), a mannitol group (MAN, Glu: 17.5 mM + MAN: 42.5 mM), a high-glucose group (HG, Glu: 60 mM), a high-glucose group combined with PU intervention (HG + PU, PU: 2.5μM/5μM/10μM), and a high-dose intervention group of PU (PU, PU: 10 μM). Each group of cells was treated for 48 h. To elucidate the molecular mechanism, we used Mitochondrial Division Inhibitor 1 (Mdivi-1; Selleck, Shanghai, China), which modulates mitophagy-related signaling. The cells were first pretreated with Mdivi-1 (5 μM) for 2 h, then co-treated with high glucose (60 mM) and high-dose PU (10 μM) for 48 h.

### 2.5. siRNA Transfection

*SESN2* siRNA, NC siRNA, and RNATransMate are all from Sangon Biotech Co., Ltd. (Shanghai, China). *SESN2* siRNA or NC siRNA was transfected into HK-2 cells at a concentration of 20 nM. HK-2 cells were inoculated in 6-well plates and transfected when the cell density reached 80%. Before transfection, the original culture medium was removed from the 6-well plate, and the serum-free culture medium was added. After a 2 h starvation period, 2.9 μL of RNATransMate was mixed with 40 pmol of siRNA, and the resulting siRNA/RNATransMate complexes were added to each well. After 8 h, this was replaced with normal medium or medium containing intervention substances, and the culture was continued for 48 h. 

### 2.6. Cell Viability Assay

The Cell Counting Kit-8 (CCK8) assay was obtained from GLpbio (Shanghai, China). A 96-well culture plate was used for cell inoculation, with each well containing 5000 HK-2 cells. After the cells fully adhered to the plate, they were cultured for 48 h in medium containing different concentrations of glucose, PU, and Mdivi-1. After culturing, the cells were rinsed once or twice with PBS, then treated with a 10% CCK8 solution. The cells were then placed in a humidified incubator for 0.5–4 h. Cell viability was normalized to the vehicle-treated control group, which was defined as 100%, and all experimental groups were expressed relative to this control.

### 2.7. Glucose Uptake Ability Test

Insulin (B.Y.T, Shanghai, China) and 2-NBDG (Glpbio, Shanghai, China) were both diluted using sugar-free culture medium. After culturing HK-2 cells for 48 h, the culture medium was discarded, and the cells were washed 1–2 times with sugar-free medium. Insulin solution (100 nM) was added, and cells were incubated at 37 °C for 1 h. After incubation, the cells were washed 1–2 times with sugar-free culture medium, and 2-NBDG solution (100 μM) was added. The cells were incubated at 37 °C in the dark for 1 h. After incubation, the cells were washed 1–2 times with sugar-free culture medium; the cells were observed under a fluorescence microscope, and photos were taken. After imaging, the cells were digested with trypsin, centrifuged, and resuspended in PBS. The fluorescence intensity of a 96-well culture plate was determined using an excitation wavelength of 485 nm and an emission wavelength of 535 nm.

### 2.8. Transmission Electron Microscopy Detection

After trypsinization and centrifugation, HK-2 cells were fixed in electron microscopy fixative for 30 min at room temperature in the dark, and then kept at 4 °C. After dehydration, soaking, and embedding, ultra-thin slices with a thickness of 70 nm were prepared, stained, and observed under a transmission electron microscope (HT7800, Hitachi, Ltd., Tokyo, Japan) for photography.

### 2.9. Fluorescent Co-Localization Measurement

HK-2 cells were seeded into a 12-well plate that had been pre-loaded with small glass disks. After the cells fully adhered to the wall, they were incubated for 48 h. Following the intervention, the culture medium was removed and the cells were rinsed two to three times with PBS. After fixation and membrane permeabilization, they were incubated at room temperature with 10% goat serum for 1 h, incubated with TOM20 (1:100, Proteintech, Wuhan, China) and LC3 (1:50, Proteintech) overnight at 4 °C, and incubated with CoraLite594-labeled goat anti-mouse IgG (1:300, Proteintech) and FITC-labeled goat anti-rabbit IgG (1:100, Boster, Wuhan, China) at room temperature in the dark for 2 h. The cell nucleus was DAPI (Boster)-stained for 5 min. An appropriate amount of anti-fluorescence-quenching PVP mounting medium (Boster) was applied for mounting. Photos were taken under a laser confocal microscope (Zeiss LSM 900 META, Jena, Germany). ImageJ software was used to analyze the fluorescence co-localization coefficient.

### 2.10. Western Blot

Renal tissue or HK-2 cells were lysed to obtain total renal and cellular proteins. The BCA method was used to quantify protein concentration. The same amount of protein was loaded and run on a 10% or 12% SDS-PAGE gel, then transferred to a PVDF membrane and sealed with 5% skimmed milk powder in a shaking bed at room temperature for 1 h. Then, it was placed in primary antibodies against KIM-1 (HA721535, 1:1000; Huabio, Hangzhou, China), SESN2 (A14220, 1:1000; Abclonal, Wuhan, China), PINK1 (A00201-2, 1:1000; Boster), Parkin (381626, 1:1000; ZenBio, Chengdu, China), P62 (BA2849, 1:1000; Boster), LC3 (14600-1-AP, 1:1000; Proteintech), caspase-1 (ET1608-69, 1:1000; HuaBio), IL-18 (10663-1-AP, 1:2000; Proteintech), FTH1 (BM4487, 1:1000; Boster), ACSL4 (22401-1-AP, 1:3000; Proteintech), β-actin (EM21002, 1:10,000; HuaBio), and GAPDH (GB15002, 1:1000; Servicebio, Wuhan, China), and incubated overnight at 4 °C. On the second day, the HRP-conjugated secondary antibody (HA1001, HuaBio; HA1006, HuaBio) was incubated at room temperature for 1 h. After cleaning, the ECL luminescence reagent (Purmei Biological Technology Co., Ltd., Wuhan, China) was added dropwise, and then the PVDF bands were imaged on a gel imager (Azure Biosystems C600; Azure Biosystems, Inc., Dublin, CA, USA). ImageJ image analysis software was used to analyze the grayscale level of bands.

### 2.11. Statistical Analysis

All numerical data collected are uniformly expressed as the mean ± standard deviation (SD). The sample size, denoted by “*n*” (number of animals or biological replicates), is precisely noted for each dataset. The Shapiro–Wilk test was applied to examine the normality of data distribution. Repeated-measures ANOVA was used to evaluate changes in body weight. For other multi-group comparisons, statistical significance was evaluated using one-way analysis of variance (one-way ANOVA), followed by the LSD *t*-test. All computations were performed using GraphPad Prism software (Version 9.0, GraphPad Software, Boston, MA, USA) and SPSS 26.0. A probability (*p*) value below 0.05 (*p* < 0.05) was deemed statistically significant.

## 3. Results

### 3.1. Effect of PU on Body Weight and Kidney Weight of Diabetic Mice

In order to explore the effect of PU on renal injury in diabetes, this study conducted an in vivo experiment in mice (Figure 1A). There was no statistically significant difference in initial body weight across the three groups. The weight of the CON group mice showed an upward trend throughout the experimental period. The body weight of mice in the DM and DM + PU groups was similar during the first 8 weeks of the experiment after high-fat diet initiation, and both were elevated compared to the CON group, but after an injection of STZ, both groups of mice began to show a decrease in body weight (Figure 1B). In comparison with the CON group, the DM group showed a marked increase in renal volume and kidney weight-to-body weight ratio (*p* < 0.05). After PU intervention, the kidney coefficient significantly decreased (*p* < 0.05) (Figure 1D,E).

### 3.2. PU Alleviates Renal Injury in Diabetic Mice

BUN and CREA are important indicators reflecting kidney function. Mice in the DM group showed significantly higher serum levels of both BUN and CREA than those in the CON group (*p* < 0.01), suggesting renal dysfunction. Compared with the DM group, the serum BUN and CREA levels of the DM + PU group mice were significantly decreased (*p* < 0.05) (Figure 2A,B). The kidney injury molecule KIM-1 is a reliable biological marker for detecting kidney injury, with only trace expression in normal kidneys. Compared with the CON group, the DM group showed a significant upregulation of renal KIM-1 protein (*p* < 0.01), suggesting kidney damage. In contrast, PU treatment (DM + PU) led to a notable reduction in KIM-1 expression relative to the DM group (*p* < 0.01) (Figure 2C).

HE staining revealed clear renal architecture and neatly arranged tubular epithelial cells in the CON group. In contrast, the DM group exhibited indistinct tubular boundaries, disorganized and irregular epithelial cell arrangement, cellular swelling, pale and loose cytoplasm, evident vacuolar degeneration, a marked increase in the renal tubular injury score (*p* < 0.01), and an elevated glomerular cell count (Figure 2D). MASSON staining showed a large amount of blue staining substance deposition in the glomerular sac and renal tubules of the DM group, with a significant increase in CVF (*p* < 0.01), indicating collagen deposition and fibrosis in renal tissue (Figure 2E). According to PASM staining, the DM group exhibited glomerulosclerosis with increased glomerular volume, mesangial matrix proliferation, and thickening of the glomerular basement membrane. Furthermore, the PASM-positive area significantly increased (*p* < 0.01) (Figure 2F). Compared with the DM group, the PU intervention significantly mitigated the above pathological lesions.

### 3.3. PU Reduces HK-2 Cell Damage in a High-Glucose Environment

To further explore the mechanism of PU in renal injury in diabetes, this study used HK-2 cells to establish a renal cell injury model in a high-glucose environment for in vitro experiments. This study used the CCK8 method to determine the appropriate concentrations of glucose, PU, and their combination (Figure 3A–C). Finally, 60 mM glucose was used in combination with 2.5, 5, and 10 μM PU for subsequent experiments, and a 42.5 mM mannitol group was set as the hypertonic control.

To examine the impact of PU on glucose metabolism in HK-2 cells cultured in a high-glucose milieu, the glucose uptake of these cells following insulin stimulation was measured using the fluorescent glucose probe 2-NBDG (Figure 3D,E). 2-NBDG enters cells through glucose transporters and exhibits green fluorescence under a fluorescence microscope. Insulin can increase the quantity and activity of glucose transporters on the cell membrane, enhancing glucose uptake capacity. Insulin stimulation increased the 2-NBDG fluorescence intensity in HK-2 cells, suggesting that these cells are responsive to insulin. Compared with the CON + Ins group, the HG + Ins group showed a substantial reduction in cellular fluorescence (*p* < 0.01), indicating decreased glucose uptake capacity under high-glucose conditions. Relative to the HG + Ins group, the 2-NBDG fluorescence intensity in cells treated with low, medium, and high doses of PU showed a progressive increase (*p* < 0.01), indicating that PU can alter insulin-stimulated glucose uptake in HK-2 cells.

To clarify the effect of PU on HK-2 cell damage under high-glucose conditions, this study detected the expression level of KIM-1 protein in HK-2 cells (Figure 3F). KIM-1 protein expression in HK-2 cells was markedly upregulated in the HG group versus the CON group (*p* < 0.01), reflecting high-glucose-induced cell injury, whereas treatment with PU at low, medium, and high doses significantly reduced KIM-1 levels compared with the HG group (*p* < 0.01).

### 3.4. PU Alleviates Pyroptosis-Related Indicators in Diabetic Mice and in HK-2 Cells Under a High-Glucose Environment

Pyroptosis is a form of programmed cell death, and abnormal levels of pyroptosis are important contributors to cell damage. This study investigated the expression of pyroptosis-associated proteins (caspase-1 and IL-18) to elucidate how PU influences pyroptosis in the kidneys of diabetic mice and in HK-2 cells cultured under high-glucose conditions (Figure 4). Significant upregulation of caspase-1 and IL-18 expression was observed in the kidneys of DM mice and in high-glucose-treated HK-2 cells relative to the CON group (*p* < 0.01). However, PU intervention markedly reduced these protein levels (*p* < 0.01), suggesting that PU may protect against renal injury by suppressing the expression of pyroptosis-related indicators.

### 3.5. PU Alleviates Ferroptosis-Related Indicators in Diabetic Mice and in HK-2 Cells Under High-Glucose Environment

Ferroptosis is a form of cell death that depends on the accumulation of iron and results in increased levels of toxic lipid peroxides. Like pyroptosis, it can also lead to renal damage in diabetes. This study examined the expression levels of ferroptosis-related proteins (FTH1 and ACSL4) (Figure 5). Compared with the CON group, the DM group showed significantly lower FTH1 expression and higher ACSL4 expression in the kidneys (*p* < 0.05), with comparable changes in high-glucose HK-2 cells. Increased FTH1 and decreased ACSL4 protein levels were observed after PU intervention (*p* < 0.05), indicating that PU might ameliorate ferroptosis-related indicators in diabetic mouse kidneys and high-glucose-induced HK-2 cells.

### 3.6. PU Alleviates Cell Pyroptosis-Related Indicators and Ferroptosis-Related Indicators in Association with Mitophagy-Related Signaling Modulation

Recent studies have found that increased levels of cell death may be related to abnormal mitophagy. To clarify the mechanism by which PU reduces pyroptosis- and ferroptosis-related indicators in the kidney of diabetic mice, this study detected the expression levels of mitophagy-related proteins (PINK1, Parkin, P62, and LC3II/I) (Figure 6A,B). Compared with the CON group, the DM group and high-glucose-treated HK-2 cells exhibited significantly reduced expression of PINK1, Parkin, and the LC3-II/I ratio (*p* < 0.05), along with a significant elevation in P62 expression (*p* < 0.01). In the PU group, the levels of PINK1, Parkin, and the LC3-II/I ratio were significantly elevated (*p* < 0.05), whereas P62 expression was markedly reduced (*p* < 0.05), suggesting that PU intervention was associated with restored mitophagy levels in the injured kidneys and cells.

To further explore whether PU modulates pyroptosis- and ferroptosis-related indicators of HK-2 cells under high-glucose conditions, potentially involving mitophagy-related signaling, this study used Mdivi-1 to inhibit the mitophagy-related proteins in cells in the HG + PU(10 μM) group, and to detect pyroptosis- and ferroptosis-related indicators in HK-2 cells (Figure 6C). Compared with the HG + PU(10 μM) group, the expression levels of PINK1, Parkin, and LC3II/I in HK-2 cells after administration of Mdivi-1 were significantly lower (*p* < 0.05), and P62 was significantly higher (*p* < 0.01). The observed changes were comparable to those seen in the HG group, suggesting that Mdivi-1 attenuated the protective effect of PU against high-glucose-induced mitophagy dysfunction in HK-2 cells.

To further explore the ultrastructural features associated with pyroptosis and ferroptosis in HK-2 cells after Mdivi-1 treatment, we examined cell ultrastructure (Figure 6D). Compared with the CON group, the HG group exhibited vesicle formation on the cell membrane, mitochondrial shrinkage, increased mitochondrial membrane density, and disruption or even loss of mitochondrial cristae. These findings were consistent with the electron microscopic features of both pyroptosis and ferroptosis. PU intervention alleviated HK-2 cell damage and improved cell membrane damage and mitochondrial structural abnormalities. However, the characteristics of cell pyroptosis and ferroptosis were apparent after the application of Mdivi-1, suggesting that PU may improve pyroptosis- and ferroptosis-related indicators in association with mitophagy-related signaling. Western blot analysis revealed that the HG + PU(10 μM) + Mdivi-1 group exhibited significantly elevated expression of caspase-1, IL-18, and ACSL4 (*p* < 0.05), along with a significant reduction in FTH1 expression (*p* < 0.05), indicating that when the level of mitophagy was suppressed, the cell pyroptosis- and ferroptosis-related indicators were increased, suggesting that PU might alleviate the high-glucose-induced HK-2 cell pyroptosis- and ferroptosis-related indicators by regulating cell mitophagy (Figure 6E,F).

### 3.7. PU Modulates Mitophagy-Related Signaling in Association with SESN2 Pathway Activation

SESN2 is a highly conserved stress-induced protein that may regulate mitophagy. Therefore, this study detected the expression of the SESN2 signaling pathway in mouse kidneys and HK-2 cells (Figure 7A,B). Compared with the CON group, the expression level of SESN2 in the kidneys of DM group mice and HK-2 cells of the HG group decreased significantly (*p* < 0.01), and increased significantly after PU intervention (*p* < 0.01), indicating that the SESN2 signaling pathway was impaired in the kidneys of diabetic mice and in high-glucose-treated HK-2 cells, and PU could improve the expression of SESN2 protein.

To further validate whether PU modulates mitophagy-related signaling in association with the SESN2 signaling pathway, *SESN2* siRNA was transfected into HK-2 cells to knock down *SESN2* expression in the HG + PU (10 μM) group, followed by assessment of mitophagy-related markers (Figure 7C). Compared with the HG + PU(10 μM) + siNC group, the expression of SESN2 in HK-2 cells was significantly decreased after transfection with *SESN2* siRNA (*p* < 0.01), indicating that the role of PU in activating the SESN2 signaling pathway in HK-2 cells in a high-glucose environment was weakened when *SESN2* siRNA was present.

TOM20 is located on the outer membrane of mitochondria and is widely used as a mitochondrial localization marker. Compared with the HG + siNC group, the HG + PU(10 μM) + siNC group showed enhanced fluorescence co-localization of TOM20 and the autophagy marker LC3 in HK-2 cells, and a significant increase in the Pearson correlation coefficient (*p* < 0.05). After transfection with *SESN2* siRNA, the co-localization of TOM20 and LC3 was weakened, and the Pearson correlation coefficient was significantly reduced (*p* < 0.05) (Figure 7D). Additionally, the expression levels of PINK1, Parkin, and LC3II/I in HK-2 cells significantly decreased (*p* < 0.05), and P62 significantly increased (*p* < 0.05), suggesting that PU could modulate mitophagy-related signaling in HK-2 cells under high-glucose stress, which was associated with SESN2 pathway activation (Figure 7E).

## 4. Discussion

DKD is a severe microvascular complication of diabetes, affecting approximately 50% of type 2 and one-third of type 1 diabetic patients, and may progress to end-stage renal disease [24]. Combining a high-fat diet with low-dose STZ treatment produces a diabetic mouse model that exhibits both the natural disease course and metabolic characteristics resembling human type 2 diabetes. The success of diabetic-related kidney injury in vivo was evaluated by endpoint indicators such as BUN and CREA [25,26]. When the kidneys are exposed to a high-glucose environment, renal tubular epithelial cells are the initial site of damage and are considered the driving force behind kidney disease [27]. In many studies on renal injury in diabetes, high-glucose cell culture is the most commonly used in vitro model [28,29]. Therefore, on the basis of previous research, this study took C57BL/6 male mice as experimental subjects to establish an in vivo model of diabetes-related renal injury through a high-fat diet combined with intraperitoneal injection of 100 mg/kg of STZ, and HK-2 cells as experimental subjects to establish a renal cell injury model in a high-glucose environment, thereby enabling both in vivo and in vitro experiments.

Current management of DKD primarily focuses on glycemic control, lipid lowering, blood pressure reduction, and the use of renin–angiotensin system (RAS) blockers, with dialysis or kidney transplantation reserved for end-stage disease. However, specific therapeutic agents are still lacking [30]. Research has shown that existing treatment methods may lead to adverse consequences such as hyperkalemia and acute kidney injury [31]. Moreover, kidney transplantation cannot avoid the recurrence of DKD, nor can it improve other complications of diabetes. Thus, natural phytochemicals with safety and efficacy, such as baicalin and apigenin, have attracted increasing attention, as they have been confirmed to exert beneficial effects against diabetic kidney disease [32,33]. In our previous research, PU not only plays a role in preventing and treating liver injury in diabetes, but also shows great potential in preventing and treating DKD [22,34]. This study found that PU can effectively improve histological changes in mouse kidneys, reduce abnormally elevated serum BUN and CREA levels, and inhibit KIM-1 protein expression. In cell-based experiments, PU was found to improve the morphology of HK-2 cells in a high-glucose environment and alter insulin-stimulated glucose uptake.

Aberrant renal cell death is now recognized as a central event in the development of diverse kidney disorders, which can inflict renal injury either directly or indirectly by recruiting immune cells and triggering inflammatory responses [35,36]. Excessive pyroptosis and ferroptosis in DKD have received extensive attention in recent years. In DKD mice, QiZhiJiangTangJiaoNang can reduce renal inflammation, inhibit the NLRP3/caspase-1/GSDMD signaling pathway by activating Nrf2, reduce podocyte pyroptosis, and improve renal injury [37]. Moreover, VX-765 treatment decreased caspase-1 expression, suppressed inflammatory cell infiltration, downregulated pyroptosis-associated proteins, and alleviated renal tubulointerstitial fibrosis [38]. In the DKD rat model induced by high-fat diets combined with STZ, Schisandrae mixture alleviates oxidative stress and inhibits GPX4/ACSL4-mediated ferroptosis by regulating the HIF-1α/HO-1 pathway [39]. The Qizhi Tongluo Formula ameliorated kidney damage in db/db mice. This effect was achieved by blocking iron overload in renal tubules, reducing oxidative stress, and limiting lipid peroxide formation [40]. In this study, the expression of caspase-1, IL-18, and ACSL4 proteins increased, and the expression of FTH1 protein decreased in the kidneys of diabetic mice and high-glucose-induced HK-2 cells, indicating that pyroptosis- and ferroptosis-related indicators increased, while PU reduced them. At the same time, growing evidence indicates that the activation of multiple pyroptosis-related signaling pathways can induce ferroptosis, and ferroptosis often co-occurs with inflammation and pyroptosis [41]. In diabetic nephropathy, enhancing the AdipoR1/AMPK signaling pathway will mitigate mitochondrial damage, thereby reducing pyroptosis and ferroptosis in the kidney [42]. Knockout of *Nlrp3*, a mouse pyroptosis-related gene, can reduce lipopolysaccharide-induced renal inflammation and ferroptosis [43]. Meanwhile, inhibition of ferroptosis can block imidacloprid-induced pyroptosis in the mouse kidney [44], suggesting that pyroptosis and ferroptosis may interact in renal injury. However, the interaction between pyroptosis and ferroptosis in DKD remains poorly understood, with much yet to be discovered.

Mitochondria serve as the signaling hub for cell death, and emerging evidence suggests that mitophagy may be involved in the pathways of pyroptosis and ferroptosis [45,46,47]. In the mouse kidney injury induced by high fat and high fructose, magnolia alkaloid can inhibit NLRP3/caspase-1-mediated pyroptosis by promoting Parkin/PINK1-dependent mitophagy, thereby improving kidney injury [48]. Germacrone—a bioactive compound with anti-ferroptosis properties—activated PINK1/Parkin-mediated mitophagy, suppressed iron deposition in high-glucose-exposed renal tubular cells, and mitigated ferroptosis via the mtDNA/cGAS/STING axis [49]. PU facilitates mitochondrial renewal by increasing the amount of newly synthesized mitochondrial proteins, which in turn promotes mitophagy and mitochondrial biogenesis, thereby maintaining mitochondrial homeostasis. Consequently, PU appears highly promising for modulating mitochondrial function [50]. Our study found that PU can upregulate mitophagy-related protein expression, including PINK1 and Parkin, in the kidneys and renal cells, and reduce the expression of pyroptosis- and ferroptosis-related proteins, including caspase-1, IL-18, and ACSL4. However, this effect was attenuated after Mdivi-1 treatment, which was accompanied by increases in pyroptosis- and ferroptosis-related indicators, indicating that enhancing the expression of key mitophagy signaling pathways could effectively reduce pyroptosis- and ferroptosis-related indicators in diabetic-related kidney injury.

SESN2 plays an important role in regulating mitochondrial function, particularly by promoting mitophagy, and can modulate mitochondrial metabolism [18]. Icariin can increase SESN2-induced mitophagy, thereby inhibiting NLRP3 inflammasome activation via the Keap1-Nrf2/HO-1 axis in DKD rats [51]. Protein–protein interaction data from the STITCH database show that caspase-3/-8/-9, Bax, Bcl-2, mTOR, and ULK1 are involved in PU-induced apoptosis and autophagy signaling pathways [52], while ULK1 overexpression leads to phosphorylation of SESN2 [53]. Therefore, this study investigated the impact of PU on the SESN2 pathway and revealed that PU was associated with SESN2 protein expression in diabetic kidneys. In an in vitro model, transfection with *SESN2* siRNA attenuated the improvement of PU on the SESN2 signaling pathway, accompanied by a reduction in PINK1/Parkin-mediated mitophagy levels. These data indicate that PU can regulate mitochondrial autophagy in kidney cells, potentially involving the SESN2 pathway, thereby protecting against diabetic-related kidney injury. In the future, this pathway may become a star target for the prevention and treatment of DKD. 

Several limitations of the present study should be acknowledged. First, this study used only HK-2 cells to establish a high-glucose-induced cell model. Primary renal tubular cells or podocytes should be included to confirm cell-type specificity. Moreover, using *SESN2*-knockout mice or molecular docking techniques could further validate the molecular mechanisms of SESN2 in DKD. Second, the dose of PU (20 mg/kg) was selected based on our previous studies. Thus, a dose–response curve and pharmacokinetic evaluation under diabetic conditions are required for translation. Third, our mitophagy assessment reflects initiation, and the inhibitory effect of Mdivi-1 on mitochondrial complex I may be a confounding factor. Furthermore, it should be noted that pyroptosis and ferroptosis were inferred from protein markers rather than functional endpoints in this study, and further relevant assays are needed in future studies. Future research should focus on: (1) further mechanistic validation using *SESN2*-deficient models, dynamic flux assays, and functional cell death endpoints, and (2) translational extension through dose–response/pharmacokinetic studies, broader cell models, and human DKD sample verification.

## 5. Conclusions

This study proposes and examines a potential mechanism linking SESN2-related mitophagy to changes in pyroptosis- and ferroptosis-related markers in diabetes-associated renal injury, and may provide novel insights into tubular injury in DKD; it also found that PU was associated with upregulation of SESN2 signaling and enhanced mitophagy-related signaling. These changes were accompanied by reductions in pyroptosis- and ferroptosis-related indicators and by improvement in diabetes-associated renal injury. In addition, our findings provide preliminary evidence for the renoprotective effect of PU against DKD, potentially involving SESN2-related mitophagy and the concurrent regulation of pyroptosis- and ferroptosis-related markers in renal cells. Collectively, PU warrants further investigation as a candidate compound for DKD.

## Figures and Tables

**Figure 1:**
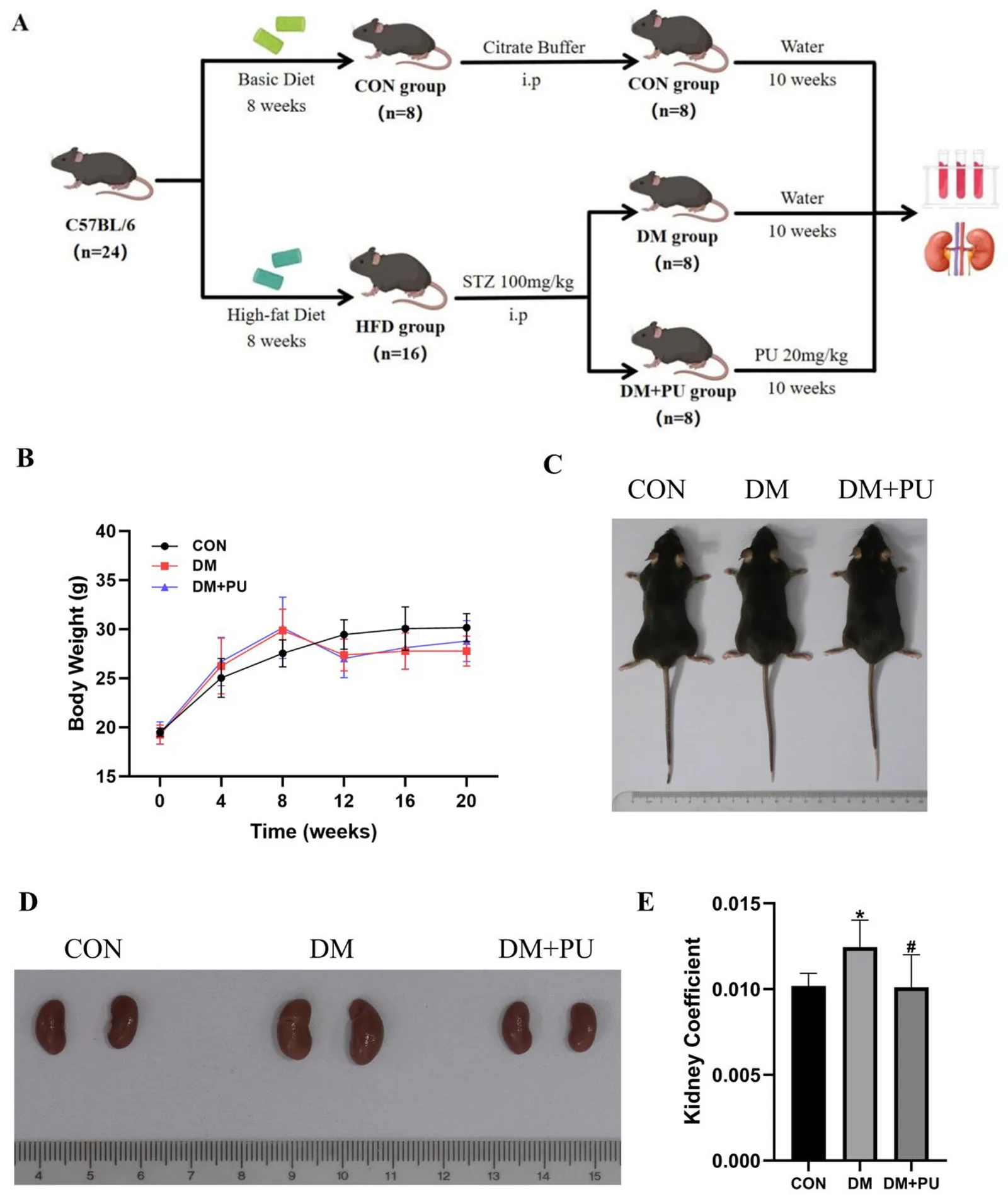
Effect of PU on body weight and kidney weight of diabetic mice. (**A**) Schematic diagram of in vivo experimental protocol. (**B**) Mouse weight change curve. (**C**) Mouse body size at week 20. (**D**) Mouse kidney morphology. (**E**) Mouse kidney coefficient (kidney weight/body weight). *n* = 6 biological replicates. * *p* < 0.05, compared with the CON group; # *p* < 0.05, compared with the DM group.

**Figure 2:**
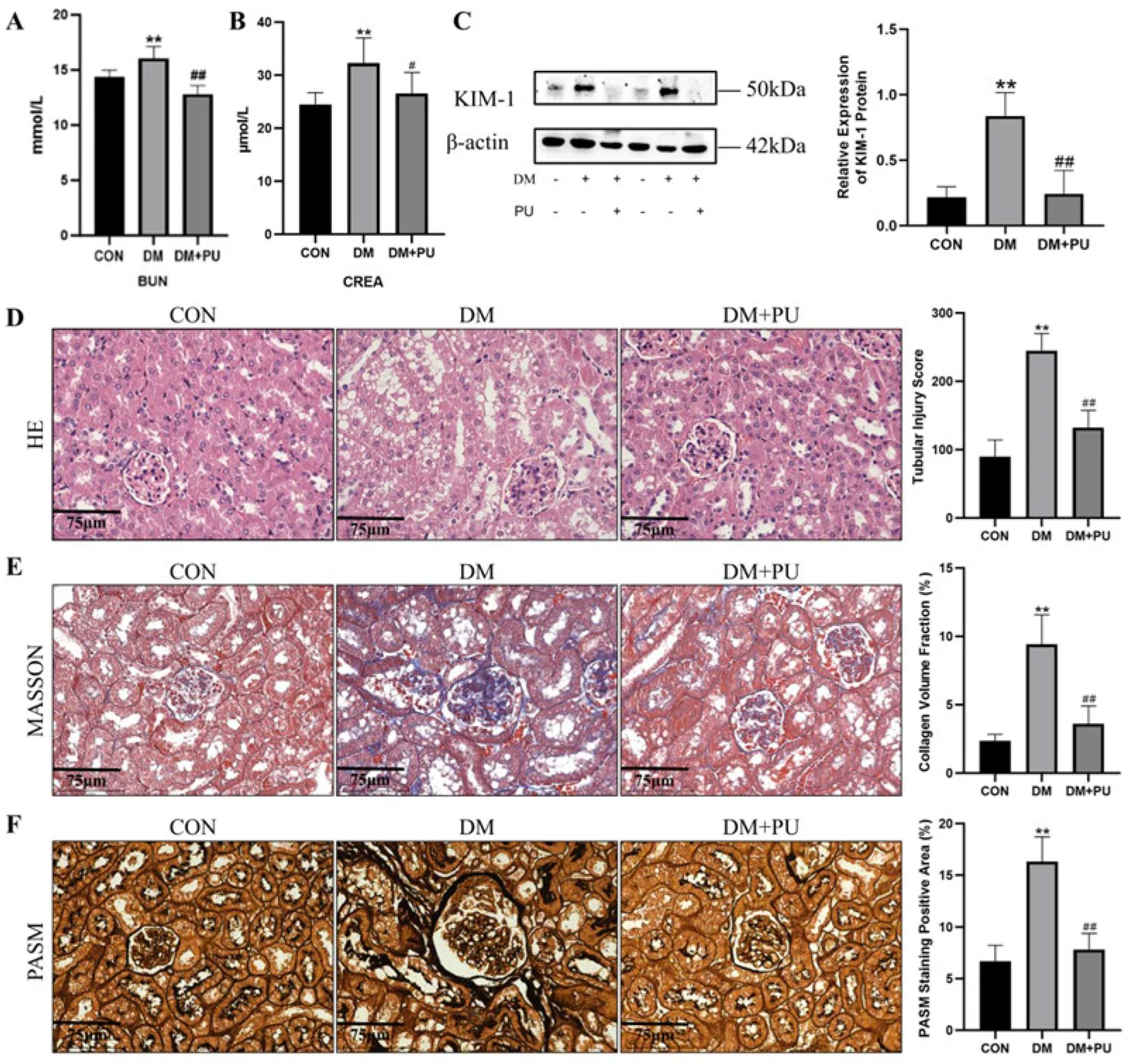
Effects of PU on renal function and renal tissue structure in diabetic mice. (**A**) Serum BUN level in mice, *n* = 6 biological replicates. (**B**) Serum CREA level in mice, *n* = 6 biological replicates. (**C**) Expression level of KIM-1 protein in mouse kidneys, *n* = 4 biological replicates. (**D**) HE staining of mouse kidneys and renal tubular injury score, *n* = 4 biological replicates. (**E**) MASSON staining of mouse kidneys and determination of CVF in each group of slices, *n* = 3 biological replicates. (**F**) PASM staining of mouse kidneys and determination of PASM-positive area in each group of sections, *n* = 3 biological replicates. ** *p* < 0.01, compared with CON group; # *p* < 0.05, ## *p* < 0.01, compared with DM group.

**Figure 3:**
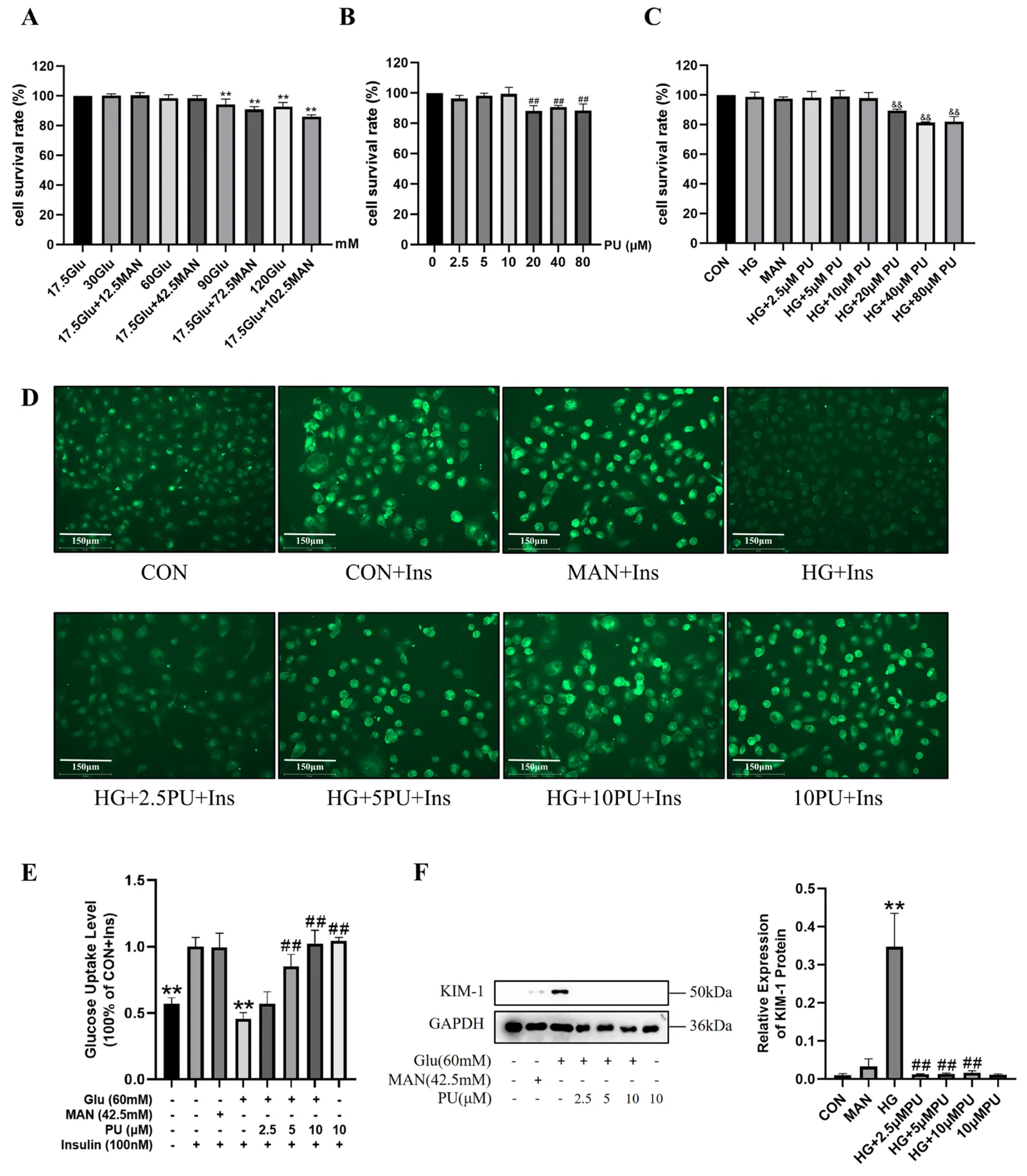
Effect of PU on the morphology and the glucose uptake ability of HK-2 cells under a high-glucose environment. (**A**) Effect of 30, 60, 90, and 120 mM glucose on HK-2 cell viability. ** *p* < 0.01, compared with the 17.5 mM Glu group. (**B**) 2.5, 5, 10, 20, 40, and 80 μM PU on HK-2 cell viability. ## *p* < 0.01, compared with the 0 μM PU group. (**C**) Effect of 60 mM glucose combined with 2.5, 5, 10, 20, 40, and 80 μM PU on HK-2 cell viability. && *p* < 0.01, compared with the CON group. (**D**,**E**) Fluorescence microscopy images of cells stained with 2-NBDG and the relative fluorescence intensity of 2-NBDG in cells measured by a fluorescence microplate reader. ** *p* < 0.01, compared with the CON + Ins group; ## *p* < 0.01, compared with the HG + Ins group. (**F**) The expression level of KIM-1 protein in HK-2 cells. *n* = 3 biological replicates. ** *p* < 0.01, compared with the CON group; ## *p* < 0.01, compared with the HG group.

**Figure 4:**
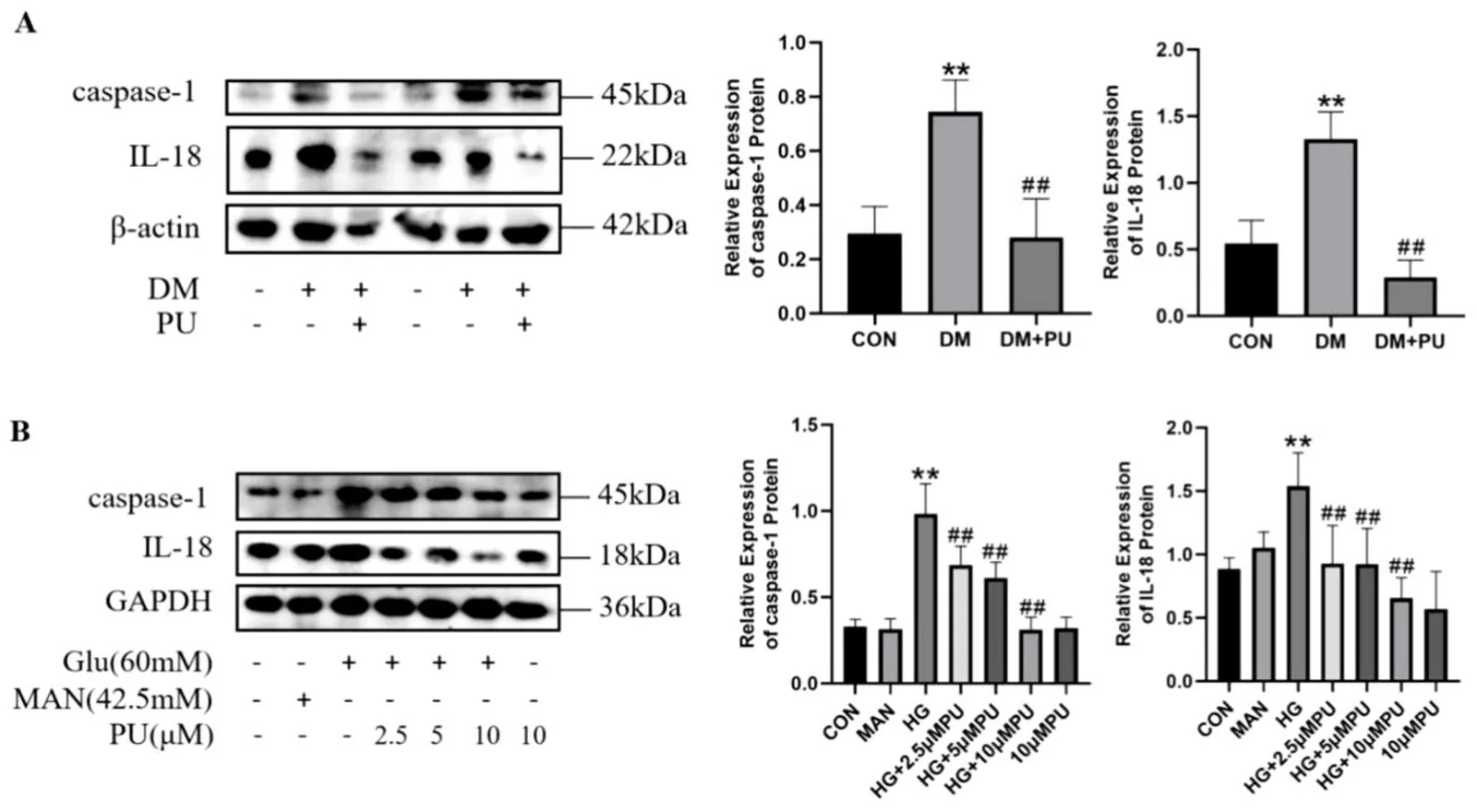
Effect of PU on pyroptosis-related indicators in the kidneys of diabetic mice and HK-2 cells in a high-glucose environment. (**A**) Expression levels of caspase-1 and IL-18 in mouse kidneys. (**B**) Expression levels of caspase-1 and IL-18 in HK-2 cells. ** *p* < 0.01, compared with the CON group; ## *p* < 0.01, compared with the DM or HG group. *n* = 3 biological replicates.

**Figure 5:**
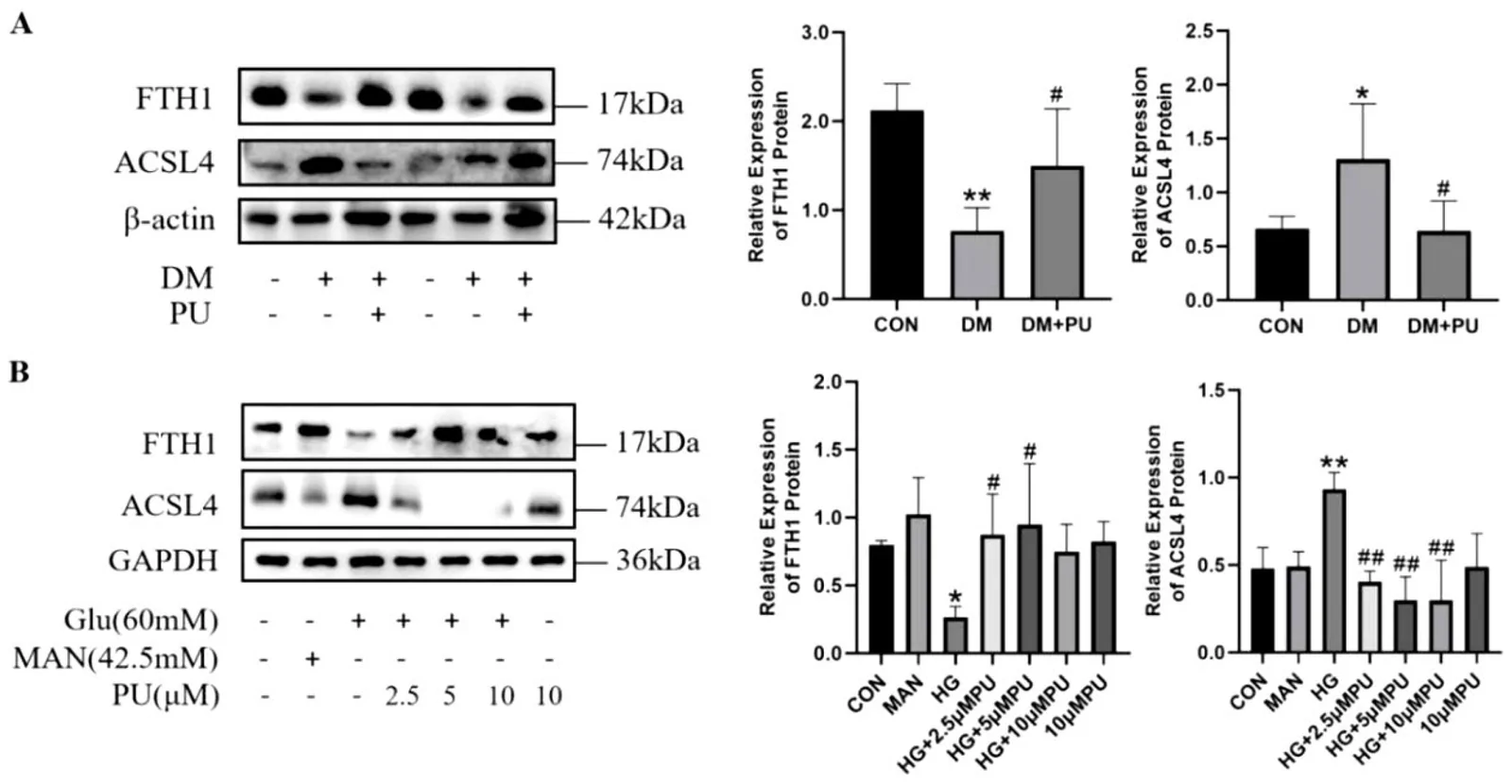
Effect of PU on ferroptosis-related indicators in the kidneys of diabetic mice and HK-2 cells in a high-glucose environment. (**A**) Expression levels of FTH1 and ACSL4 in mouse kidneys. (**B**) Expression levels of FTH1 and ACSL4 in HK-2 cells. *n* = 3 biological replicates. * *p* < 0.05, ** *p* < 0.01, compared with the CON group; # *p* < 0.05, ## *p* < 0.01, compared with the DM, or HG group.

**Figure 6:**
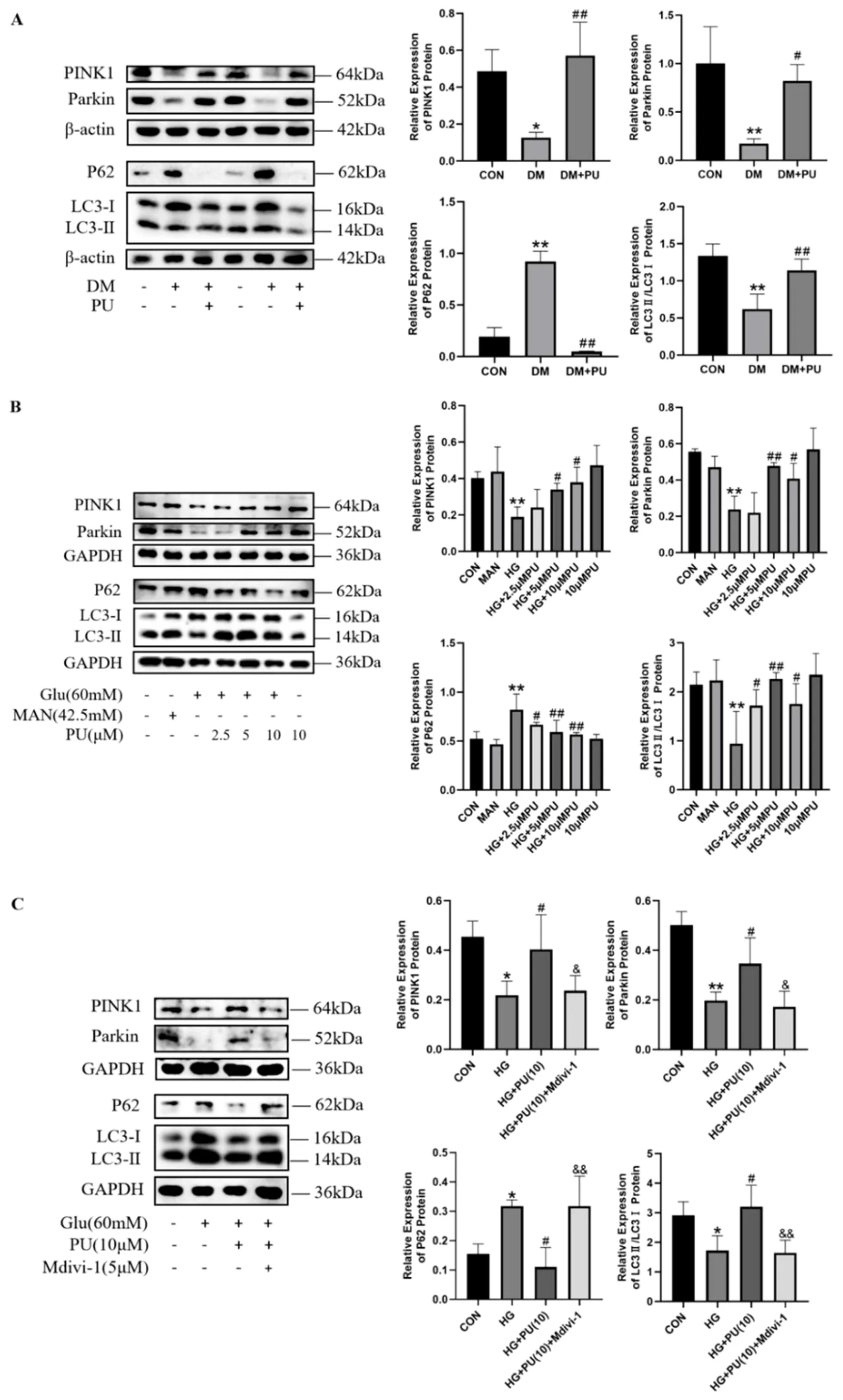

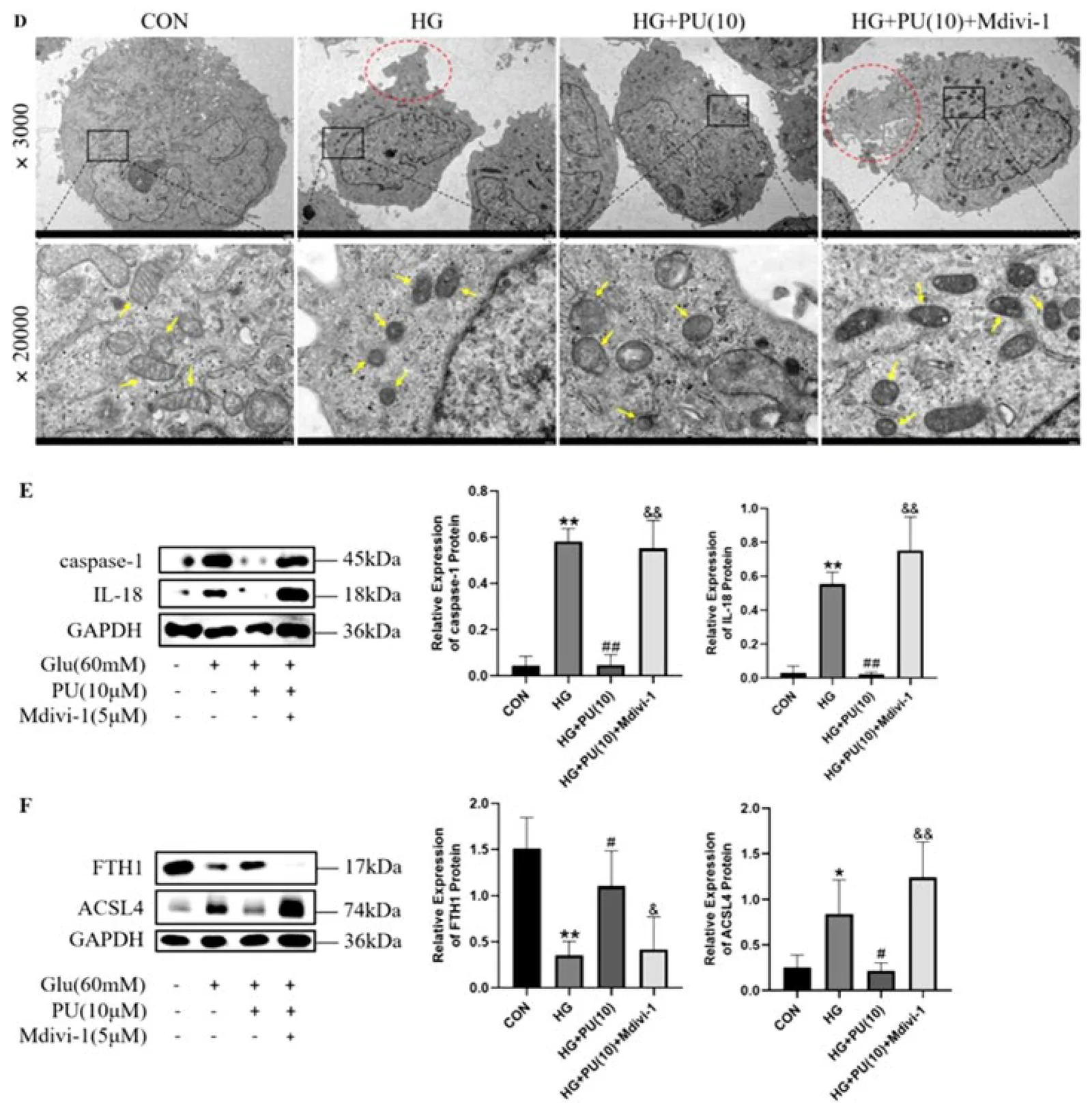
The effect of PU on mitophagy in the kidneys of diabetic mice and HK-2 cells under a high-glucose environment. (**A**) The expression levels of PINK1, Parkin, P62, and LC3II/I in mouse kidneys. (**B**) The expression levels of PINK1, Parkin, P62, and LC3II/I in HK-2 cells. (**C**) After the application of Mdivi-1, the expression levels of PINK1, Parkin, P62, and LC3II/I in HK-2 cells were measured. (**D**) HK-2 cells captured by transmission electron microscopy after application of Mdivi-1. The red coil indicates membrane vesicles, while the yellow arrow represents mitochondria. (**E**) After the application of Mdivi-1, the expression levels of caspase-1 and IL-18 in HK-2 cells were measured. (**F**) After the application of Mdivi-1, the expression levels of FTH1 and ACSL4 in HK-2 cells were measured. *n* = 3 biological replicates. * *p* < 0.05, ** *p* < 0.01, compared with the CON group; # *p* < 0.05, ## *p* < 0.01, compared with the DM or HG group; & *p* < 0.05, && *p* < 0.01, compared with the HG + PU(10 μM) group.

**Figure 7:**
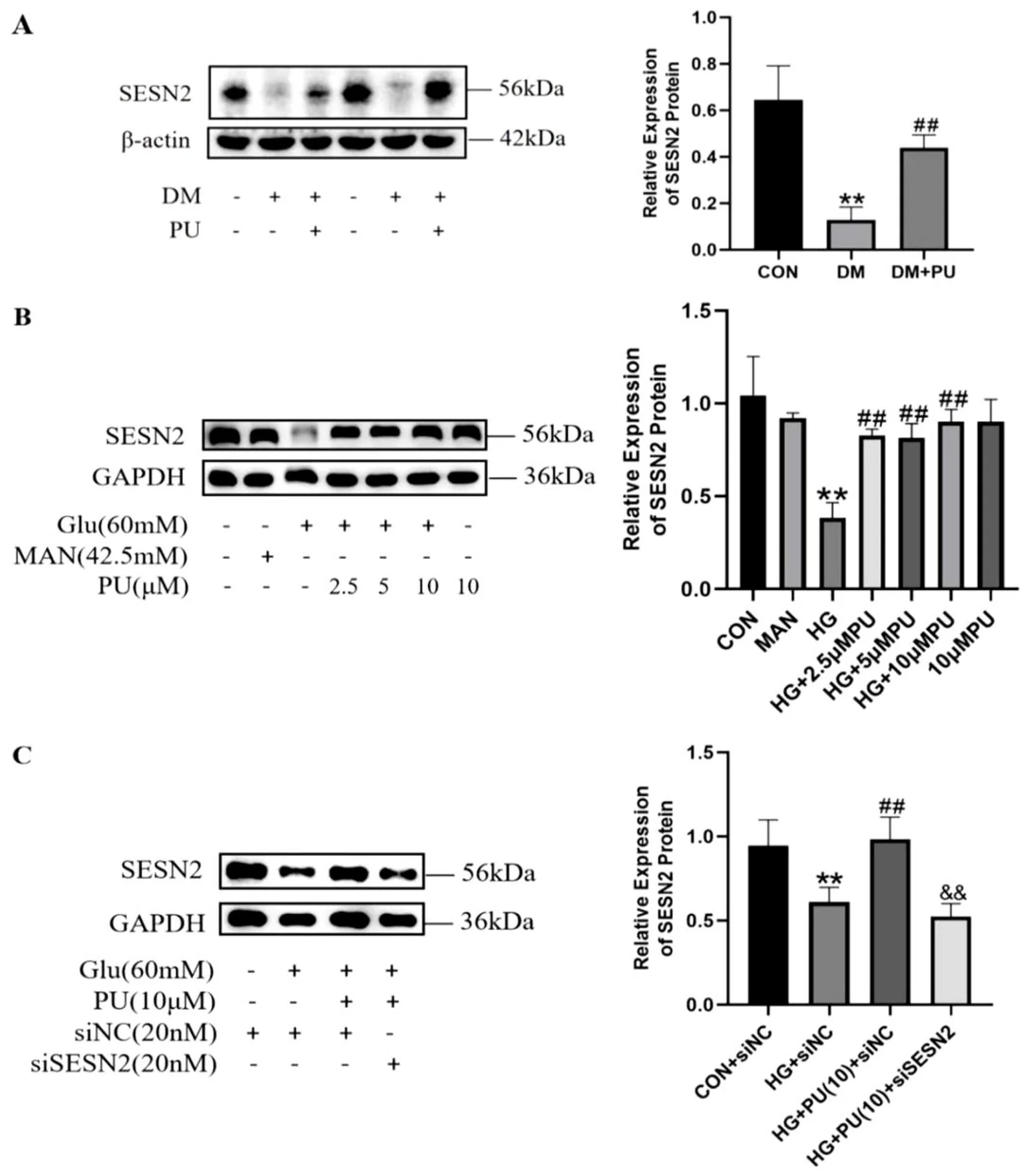

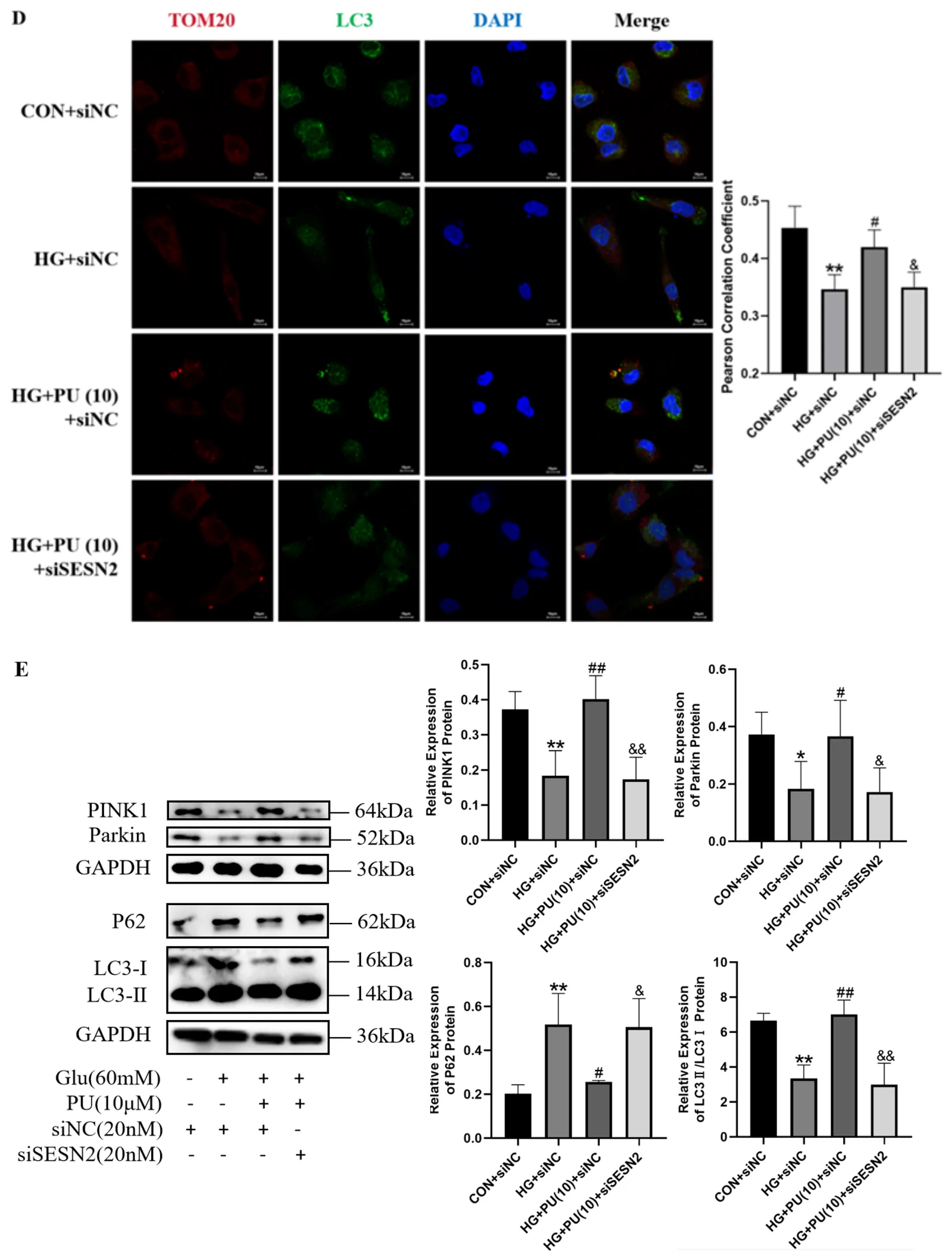
The effect of PU on the SESN2 signaling pathway in the kidneys of diabetic mice and in HK-2 cells under a high-glucose environment. (**A**) SESN2 expression level in mouse kidneys. (**B**) SESN2 expression level in HK-2 cells. (**C**) After transfection with *SESN2* siRNA, the expression level of SESN2 in HK-2 cells was measured. (**D**) Fluorescence co-localization images and correlation analysis of mitochondrial marker protein TOM20 and autophagy-related protein LC3 in HK-2 cells after transfection with *SESN2* siRNA. (**E**) After transfection with *SESN2* siRNA, the expression levels of PINK1, Parkin, P62, and LC3II/I in HK-2 cells were measured. *n* = 3 biological replicates. * *p* < 0.05, ** *p* < 0.01, compared with CON or CON + siNC group; # *p* < 0.05, ## *p* < 0.01, compared with the DM, HG, or HG + siNC group; & *p* < 0.05, && *p* < 0.01, compared with the HG + PU(10 μM) + siNC group.

## Data Availability

The original contributions presented in this study are included in the article.

## Supplementary Materials

The following supporting information can be downloaded at https://www.mdpi.com/article/10.3390/nu18193192/s1, Table S1. Scoring criteria for renal tubular injury.

## Abbreviations

ACSL4	Acyl-CoA Synthetase Long-Chain Family Member 4
BUN	Blood Urea Nitrogen
caspase-1	Cysteinyl Aspartate Acid Specific Protease-1
CREA	Creatinine
CVF	Collagen Volume Fraction
DKD	Diabetic Kidney Disease
DM	Diabetes Mellitus
FBG	Fasting Blood Glucose
FTH1	Ferritin Heavy 1
Glu	Glucose
GPX4	Glutathione Peroxidase 4
HG	High Glucose
HK-2	Human Kidney Proximal Tubular Epithelial Cells
IL-18	Interleukin-18
IL-1β	Interleukin-1 Beta
MAN	Mannitol
NLRP3	NOD-Like Receptor Thermal Protein Domain-Associated Protein 3
Nrf2	Nuclear Factor Erythroid 2-Related Factor 2
PINK1	PTEN-Induced Putative Kinase 1
PU	Punicalagin
STZ	Streptozotocin
