# Supplementary material for: Punicalagin Alleviates Diabetic Kidney Injury: Potential Involvement of SESN2-Related Mitophagy and Changes in Pyroptosis-/Ferroptosis-Related Indicators
Source: Nutrients. 2026 Sep 27;18(19):3192. doi: 10.3390/nu18193192 (PMC13667158; doi:10.3390/nu18193192)
Supplement: Supplementary file 1 [file nutrients-18-03192-s001.zip › nutrients-4530646-supplementary.pdf]

Table S1. Scoring Criteria for Renal Tubular Injury

histological features	score
presence and extent of tubular epithelial cell flattening	1
brush border loss	1
cell membrane bleb formation	1 or 2
interstitial edema	1
cytoplasmic vacuolization	1
cell necrosis	1 or 2
tubular lumen obstruction	1 or 2

HE-stained sections were scored using the Paller method to assess the degree of renal tubular injury.
